# In vivo human adipose-derived mesenchymal stem cell tracking after intra-articular delivery in a rat osteoarthritis model

**DOI:** 10.1186/s13287-016-0420-2

**Published:** 2016-11-10

**Authors:** Meng Li, Xuan Luo, Xiaoteng Lv, Victor Liu, Guangyu Zhao, Xue Zhang, Wei Cao, Richard Wang, Wen Wang

**Affiliations:** 1 Cellular Biomedicine Group, 333 Guiping Road, Shanghai, 200233 China; 2 Cellular Biomedicine Group, 19925 Stevens Creek Blvd, Suite 100, Cupertino, CA 95014 USA; 3 Plastic Surgery Hospital (Institute), Peking Union Medical College, Chinese Academy of Medical Sciences, 33 Badachu Road, Shijingshan District, Beijing, 100144 China

**Keywords:** Adipose-derived mesenchymal stem cells, Biodistribution, Intra-articular injection, Carbocyanine dyes, Osteoarthritis

## Abstract

**Background:**

Human adipose-derived mesenchymal stem cells (haMSCs) have shown efficacy in treating osteoarthritis (OA) both preclinically and clinically via intra-articular (IA) injection. However, understanding the mode of action of the cell therapy has been limited by cell tracking capability and correlation between the pharmacokinetics of the injected cells and the intended pharmacodynamics effect. This study aims to explore methodology and to understand in vivo biodistribution of clinical-grade haMSCs labeled with fluorescent dye and injected into an immunocompetent OA rat model.

**Methods:**

haMSCs labeled with fluorescent dye were investigated for their proliferation and differentiation capabilities. Labeled cells were used to establish detection threshold of a noninvasive biofluorescent imaging system before the cells (2.5 × 10^6^) were injected into a conventional rat OA model induced by medial meniscectomy for 8 weeks. We attempted to reveal the existence of labeled cells in vivo by imaging and a molecular biomarker approach, and to correlate with the in vivo efficacy and physical presence over a follow-up period up to 10 weeks.

**Results:**

In vitro proliferation and differentiation of haMSCs were not affected by the labeling of DiD dye. Detection thresholds of the labeled cells in vitro and in vivo were determined to be 10^4^ and 10^5^ cells, respectively. When 2.5 × 10^6^ haMSCs were injected into the joints of a rat OA model, fluorescent signals (or >10^5^ cells) lasted for about 10 weeks in the surgical knee joint at the same time as efficacy was observed. Signals in nonsurgical rats only lasted for 4 weeks. The human MSCs were shown to engraft to the rat joint tissues and were proliferative. Human *FOXP2* gene was only detected in the knee joint tissue, suggesting limited biodistribution locally to the joints.

**Conclusions:**

The current study represents the first attempt to correlate cell therapy efficacy on OA with the physical presence of the injected haMSCs in the OA model, and demonstrates that human adipose-derived mesenchymal stem cells persisted for 10 weeks locally in the rat joint, coinciding with the efficacy observed. It is postulated that persistence and/or proliferation of the haMSCs in the joint is required in order to exert their functions on promoting joint regeneration and/or cartilage protection, further supporting the safety and feasibility of IA injection of MSCs for the treatment of OA patients.

## Background

In the past decade, the therapeutic potential of mesenchymal stem cells (MSCs) for the treatment of osteoarthritis (OA) has been explored preclinically [1, 2]. The successful preclinical studies have led to the initiation of a number of clinical trials. In a randomized, double-blinded, controlled clinical trial [3], 24 % of a total of 55 patients who underwent a partial medial meniscectomy followed by intra-articular (IA) injection with 5–15 × 10^7^ allogeneic bone marrow-derived MSCs achieved a 15 % increase in the meniscal volume determined by magnetic resonance imaging (MRI) at the 2-year follow-up, suggesting evidence of meniscus regeneration after MSC treatment. Another proof-of-concept clinical trial in which OA patients (*n* = 9) treated with IA injection of 10 × 10^7^ adipose-derived autologous MSCs demonstrated improved Western Ontario and McMaster Universities Osteoarthritis Index (WOMAC) scores and regeneration of hyaline-like cartilage in the affected joint [4]. Recently, allogeneic bone marrow-derived MSCs were also used in an OA clinical trial. In a randomized controlled trial, 10 × 10^7^ allogeneic MSCs were delivered into OA knee joint of 15 patients. After 12 months of follow-up, both the visual analogue scale (VAS) and WOMAC pain scores, and also the WOMAC general and Lequesne scores were significantly improved when compared to the hyaluronic acid (HA) delivery control group [5]. All these clinical data indicated that MSC via IA injection may serve as a new therapeutic strategy and technology for OA treatment.

From a pharmacological perspective, it is very important to investigate the pharmacokinetics (PK)/pharmacodynamics (PD) relationship of an active therapy. Although MSC is not a traditional drug, and preclinical data generated from MSC may not always be as instructive as for drug molecules, investigating the PK/PD of MSC is essential to understand the dosing, scheduling, and mode of action (MOA) in vivo. The successful product launch of MSC as a “drug” and its translation to the clinical application for OA treatment is currently obstructed partly because of two reasons: 1) lack of specific MOA biomarkers; and 2) lack of a pharmacokinetic tool for measuring living cells due to the difficulty of in vivo cell fate tracking. Up to now, most preclinical studies on MSC PK/PD after IA injection for OA treatment have been performed by ex vivo histological analysis of β-galactosidase expression or green fluorescent protein (GFP) in the grafted cells. Horie et al. [6] used transgenic rats expressing dual luciferase (Luc) and LacZ to ascertain the Luc/LacZ^+^ synovium-derived MSCs. Twelve weeks after IA injection, regenerated menisci expressing type II collagen were found to be LacZ^+^, and the LacZ^+^ MSCs were not discovered in any other organs except for articular synovium. Murphy et al. [1] found that, after IA injection of retroviral GFP-transduced MSCs into a caprine OA model, GFP-MSCs were presented and integrated into the cell layers lining joint structures such as the meniscus, synovial capsule, and periosteum on the medial condyle 1 week after injection. Moreover, another study also found that human MSCs labeled with carboxyfluorescein diacetate succinimidyl ester (CFDA-SE) were distinguished within the cartilage at 1 week post-transplantation in a guinea pig spontaneous OA model, and labeled cells gradually disappeared from the cartilage at 3–5 weeks post-transplantation [7]. However, disadvantages of ex vivo histology analyses are obvious since the monitoring of cell fate is noncontinuous and sections of joint samples may miss some meaningful signals, especially when labeled MSCs scattered into different tissues within a joint. Furthermore, ex vivo histology analyses find it difficult to provide overall information on MSCs migrating into other distant organs in order to eliminate safety concerns for MSCs via local injection. All these shortcomings prompted development of new methods to monitor MSC PK/PD. Recently, the development of in vivo noninvasive imaging methodologies has provided one kind of tool to examine delivery, grafting, distribution, and survival of MSCs after IA injection [6, 8, 9]. However, detection sensitivity or threshold of MSC numbers that could be detected in vivo has not been fully investigated in these noninvasive imaging methodologies.

DiD is an analog of DiI (dialkylcarbocyanines) with markedly red-shifted fluorescence excitation (644 nm) and emission (665 nm) spectra. This characteristic is useful in avoiding autofluorescence and phototoxic effects as well as offering two-color labeling [10]. Recently, DiI-labeled bone marrow stromal cells were transplanted subcutaneously in nude mice to explore the in vivo traceability, and data demonstrated that DiI fluorescent signals could last at least 6 weeks after transplantation [11]. Here, we investigated the persistence and biodistribution of DiD-labeled human adipose-derived MSCs (haMSCs) after IA injection in an immunocompetent rat OA model in order to systematically understand the mechanism of MSCs in treating OA. The extended observations of human cell engraftment, biodistribution, and proliferation were reported over time in the animal model, which correlated with efficacy, and supported further development of the technology for OA therapy.

## Methods

### DiD labeling of haMSC and characterization by flow cytometry

haMSC isolation was described previously [2]. haMSC (1 × 10^6^; passage 3) were incubated with 10 μM DiD fluorescent dye (Life Technologies & Molecular Probes, USA) for 50 min at 37 °C according to the manufacturer’s instructions. Features of haMSCs labeled with or without DiD were analyzed using human MSC analysis kit (BD Biosciences, USA). hMSC Positive Cocktail (CD90 FITC, CD105 PerCP-Cy5.5 and CD73 APC) and PE hMSC Negative Cocktail (CD34, CD11b, CD19, CD45, HLA-DR) in the kit were used, while CD73 was excluded as the emission spectra of APC overlapped with that of DiD. hMSC Positive Isotype Control Cocktail (mIgG1κ FITC, mIgG1 κ PerCP-Cy5.5, and mIgG1 κ APC) and PE hMSC Negative Isotype Control Cocktail in the kit (mIgG1 κ PE and mIgG2a κ PE) were also included as an isotype control. Cell fluorescence was evaluated by flow cytometry in a FACS Calibur instrument and data were analyzed by CellQuest software (Becton Dickinson).

### Cell proliferation and differentiation assay

Proliferation of DiD-labeled or unlabeled cells was measured using the Cell Counting Kit-8 (Dojindo Molecular Technologies, USA). Differentiation of DiD-haMSCs was performed as previously reported [2]. Briefly, for adipogenic and osteogenic differentiation, cells were seeded and grown in StemPro® adipogenesis differentiation medium and StemPro® osteogenesis differentiation medium (Gibco, USA) for 3 weeks, respectively. For chondrogenic differentiation, micromass culture was used and cells were cultured in StemPro® chondrogenesis differentiation medium (Gibco, USA) for 4 weeks. The differentiation media were re-fed every 3 days. Subsequently, Oil Red O staining, Alizarin Red S staining, and Alcian Blue staining were performed to visualize the lipid droplet, calcium deposition, and cartilage, respectively. Cells were harvested at the end point of tri-lineage differentiation for real time polymerase chain reaction (PCR) to assess the specific gene expression of *ADIPONECTIN* (5′-CGTGATGGCAGAGATGGCACT-3′ for forward and 5′-GCGAATGGGTACATTGGGAACAG-3′ for reverse), *OSTEOCALCIN* (5′-TCCAAGCAGGAGGGCAATAAG-3′ for forward and 5′-GCGTTTGTAGGCGGTCTTCAAG-3′ for reverse), and *COLLAGEN TYPE 2 ALPHA 1 (COL2α1)* (5′-TCGCACTTGCCAAGACCTGAA-3′ for forward and 5′-GGTCTCTCCAAACCAGATGTG-3′ for reverse) associated with adipogenesis, osteogenesis, and chondrogenesis, respectively.

### In vitro and in vivo biofluorescent imaging

To establish the in vitro detection threshold, 100 μL phosphate-buffered saline (PBS) suspension containing different doses of DiD-haMSCs (10^6^, 10^5^, 10^4^, 10^3^, and 0 cells) were imaged by a scan of pre-warmed (37 °C) IVIS Spectrum (PerkinElmer, USA). In vivo detection sensitivity was explored by scanning nonsurgery rats immediately after IA injection with DiD-haMSCs at the same dosages (10^6^, 10^5^, 10^4^, 10^3^, and 0 cells in 100 μL PBS, respectively) as used in the in vitro study. Before imaging, the rats were anesthetized by isoflurane, and hairs were removed to reduce autofluorescence. The wavelengths of absorption and excitation were set up at 640 nm and 668 nm, respectively. The longitudinal changes in fluorescent intensity were obtained every week postinjection. Data were analyzed using the Living Image software (PerkinElmer, USA) to evaluate the average signal intensity of regions of interest (ROI). The lowest signal was adjusted to the level of autofluorescence background.

### Animal model and study design

Male Sprague-Dawley rats (250–350 g, Slac Laboratory Animal, China) were used (*n* = 18) in all of the experiments described, and were divided randomly into three groups according to the subsequent IA injections which they received: the nonsurgery group (*n* = 6), and the surgery group (*n* = 12)—itself divided into two subgroups: the free DiD group as a control (*n* = 6) and the DiD-haMSC group (*n* = 6). Experimental protocols were approved by the Ethical Committee of National Engineering Research Center of Tissue Engineering of China. OA was induced by medial meniscectomy of the right knee according to previous methods under general anesthesia and sterile conditions [12]. Eight weeks after surgery, 2.5 × 10^6^ DiD-haMSCs suspended in 100 μL PBS were injected into the right knee joint of OA rats with 26-gauge needles while the same amount of DiD-free particles and DiD-haMSCs were introduced into the right knee joint of OA rats and nonsurgery rats as control groups, respectively.

### Quantitative real-time PCR

At 14 days and 70 days postinjection, rats were sacrificed and half of the heart, liver, spleen, lung, kidney, brain, muscle adjacent to the joint, and the whole injected knee joint were collected and dissociated using an Ultra-Turrax T-50 homogenizer. DNA of the samples was isolated from 200 μL of tissue suspension using DNeasy Blood and Tissue kit (Qiagen, USA) and quantified using NanoDrop spectrophotometer (Labtech, USA). Real-time PCR was conducted to measure the level of human-specific *FOXP2* gene from a 33-ng DNA specimen in 10 μL PCR reagent mixture containing 200 nM primers and 200 nM probe. The primers and probe were as follows: 5-TGGTAGTCTGGAACACCGTAAGAGT-3 (forward); 5-CATATGGCAGGCTTTAGGTACCC-3 (reverse) for human *FOXP2*; 5-FAM-CTGGTGGGCTAAAAGGAAGAAAGAGGTC-TAMRA-3 (probe). PCR conditions were as follows: 95 °C for 5 min, followed by 40 cycles at 95 °C for 5 s, and then 60 °C for 30 s. A standard curve was generated by serial dilution of human genomic DNA from haMSCs into DNA from rat adipose-derived mesenchymal stem cells (raMSCs) (the total DNA amount was kept constant at 33 ng; Table 1). According to the standard curve, the percentage of human DNA in 33 ng total DNA from each organ or tissue was calculated.

**Table 1:** Human and rat DNA mix in quantitative real-time PCR

Total DNA in reaction		Percentage of human DNA (%)	Ct *FOXP2*
Human DNA (ng)	Rat DNA (ng)		
33	0	100	25.6700
6.6	26.4	20	28.3400
1.32	31.68	4	30.6300
0.264	32.736	0.8	33.1200
0.0528	32.9472	0.16	35.9300
0.01056	32.98944	0.032	38
0.002112	32.997888	0.0064	38
0	33	0	38

### Histological assessment

At 70 days postinjection, distal femurs and proximal tibias were excised and fixed in 4 % paraformaldehyde and decalcified in EDTA-buffered saline solution (pH 7.4, 0.25 mol/L). To assess haMSC localization and proliferation, paraffin-embedded serial sections were used for immunostaining using monoclonal antihuman mitochondria antibody (Millipore; 1:80) and monoclonal antihuman ki67 antibody (Abcam; 1:100). Human skin tissue and human hepatocellular carcinoma cell line HepG2 were used as controls, respectively. To assess haMSC efficacy, the specimens were paraffin embedded and sectioned for hematoxylin/eosin (HE) and Safranin-O/Fast Green staining. Evaluations of general morphology and proteoglycans were performed by two blinded researchers unaware of the treatment, and scored according to the Modified O’Driscoll grading system [13]. Furthermore, quantitative assessment of cartilage thickness (CT) was carried out according to a previous investigation [14].

### Statistical analysis

Statistical analysis was conducted with GraphPad Prism (version 6.0). Numerical results are presented as the mean ± SEM. One-way analysis of variance was applied to determine the difference of the optical density (OD) value, DNA expression, and signal intensity in various dosages, the Mann-Whitney *U* test was used to compare groups at each time point in the tracking image. *P* values <0.05 were considered statistically significant.

## Results

### Characteristics and viability of haMSCs by DiD labeling remained stable in vitro

**Fig. 1:**
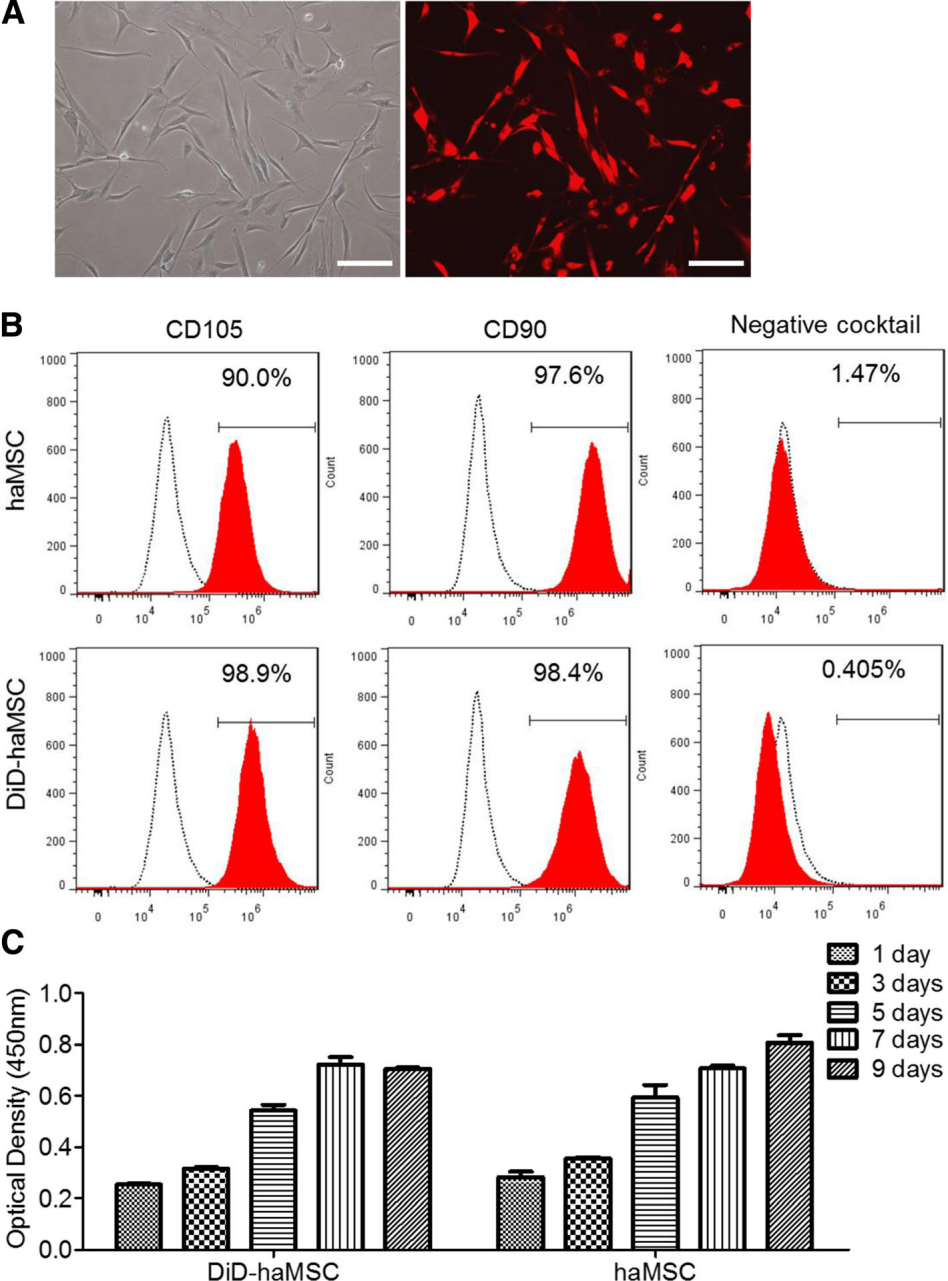
Characterization of DiD-labeled human adipose-derived mesenchymal stem cells (*haMSCs*) showing the labeling efficiency and unaltered surface markers and proliferation in vitro. **a** The high efficiency of DiD labeling, comparing cells positive in the fluorescent image with those in the bright field (*scale bars* = 50 μm). **b** After labeling, haMSCs were positive for CD90 and CD105, and negative for the cocktail including CD34, CD11b, CD19, CD45, and HLA-DR, the same as the unlabeled haMSCs. **c** Cell viability assay by OD measurement with CCK8 kit confirmed no significant variation in proliferation capacity of labeled haMSCs at days 1, 3, 5, 7, and 9 compared with unlabeled haMSCs (no statistical significance)

In order to know the labeling efficiency, haMSCs isolated from a donor via lipoaspirates and cultured to passage 4 were stained with 10 μM DiD solutions. We found that nearly all haMSCs were DiD-positive (Fig. 1a) consistent with previous investigation [11]. For the next step, we wanted to know if DiD labeling changed haMSC characteristics or not. By using flow cytometry we found that stemness of haMSCs remained after DiD labeling. Unlabeled and labeled haMSCs both were positive for CD105 (90 % and 98.9 %, respectively) and CD90 (97.6 % and 98.4 %, respectively), the main cell surface markers for MSCs. In addition, unlabeled and labeled haMSCs were negative for CD34, CD11b, CD19, CD45, and HLA-DR cocktail (1.47 % and 0.405 %, respectively), which are nonMSC markers as defined by the International Society for Cell Therapy (Fig. 1b). CCK8 assay confirmed no significant variation in proliferation capacity of labeled haMSCs at days 1, 3, 5, 7, and 9 compared with unlabeled haMSCs, indicating that DiD labeling produced no alteration on haMSC proliferation (Fig. 1c).

### DiD labeling displayed no influence on haMSC stemness in vitro

**Fig. 2:**
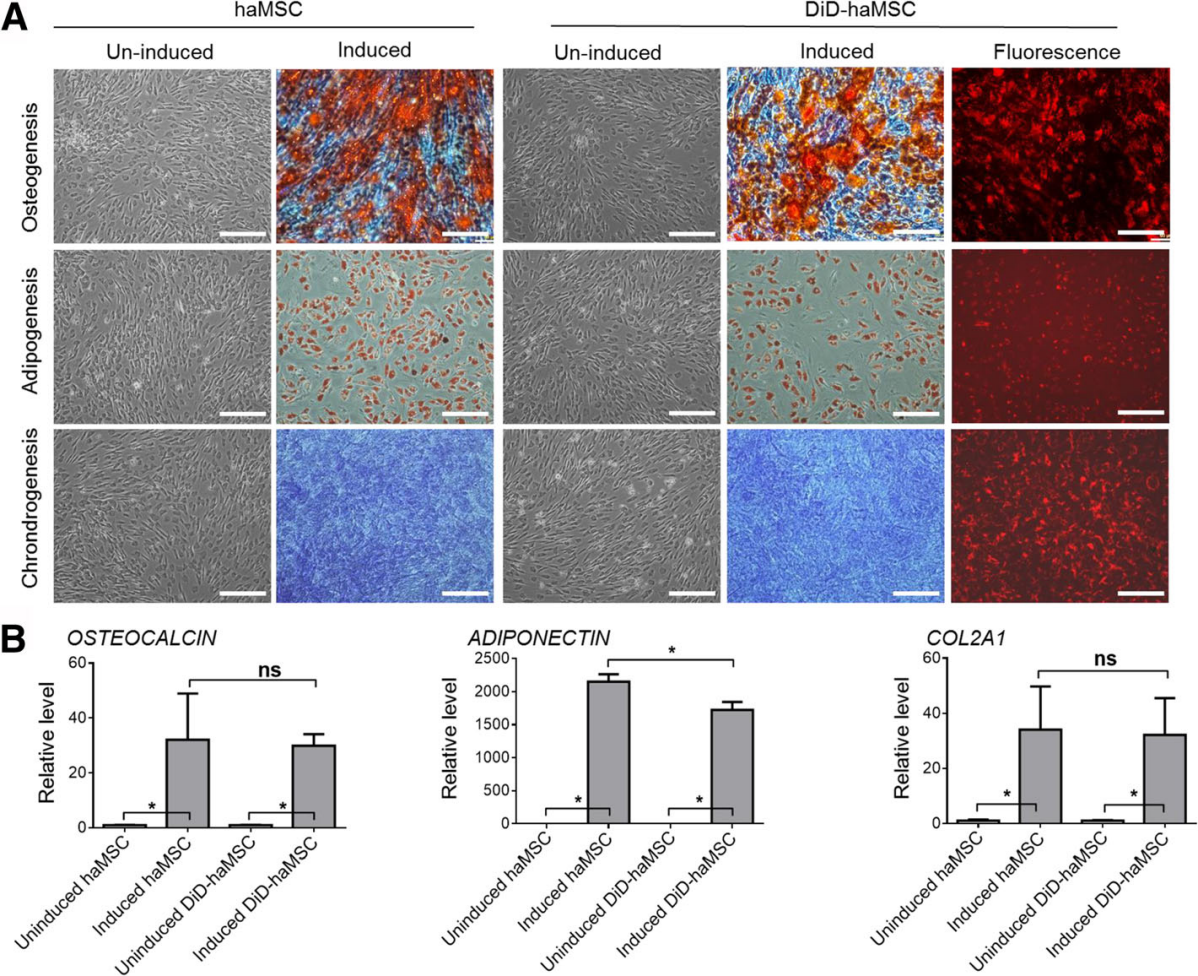
DiD labeling had no influence on differentiation of human adipose-derived mesenchymal stem cells (*haMSCs*) in vitro. **a** Unlabeled and labeled haMSCs differentiated similarly under osteogenic, adipogenic, and chondrogenic conditions, producing robust mineralized matrix-large lipid droplets, and condensing chondrocytes detecting by Alizarin Red S staining, Oil Red O staining, and Alcian Blue staining, respectively. Differentiated haMSCs retained the DiD label, as fluorescence was detected at the end of each differentiation (*scale bar* = 100 μm). **b** At the end of culturing, the osteogenic, adipogenic, and chondrogenic gene markers, *OSTEOCALCIN*, *ADIPONECTIN*, and *CAL2A1* in labeled haMSCs were upregulated during osteogenesis, adipogenesis, and chondrogenesis, respectively. No significant difference was seen between the labeled and unlabeled haMSCs for the differentiation potential, except for the adiponectin expression where labeled haMSCs showed slightly less expression than the unlabeled cells. The *P* values were obtained using one-way ANOVA analysis of variance (*P < 0.05). Data were collected from three repetitions (*n* = 3). *ns* nonsignificant

Since the characterization and proliferation of the DiD-labeled haMSCs remained unaltered, we wanted to know whether DiD labeling changed the differentiation capability of haMSCs. In vitro differentiation studies on the osteoblast, adipocyte, and chondrocyte cell lineages were carried out to compare the multipotency of unlabeled and labeled haMSCs, respectively. For osteoblast differentiation, calcium deposition and Alizarin Red staining could be observed in DiD-labeled and unlabeled haMSCs cultured in the differentiation media containing ascorbic acid and β-glycerol phosphate, while no mineralization was detected when haMSCs were grown under nonosteogenic conditions (Fig. 2a). Real-time PCR was carried out on day 21 of osteoblast differentiation (Fig. 2b). *OSTEOCALCIN* gene expression was substantially upregulated during differentiation. For adipocyte differentiation, lipid droplets could be identified by Oil Red O staining while labeled haMSCs grown in the absence of adipogenic inducers exhibited no lipid droplets (Fig. 2a). The adipogenic marker *ADIPONECTIN* was considerably upregulated during differentiation (Fig. 2b). Although DiD-labeled haMSCs have a slight decrease in *ADIPONECTIN* expression during adipogenesis compared to the unlabeled haMSCs, *ADIPONECTIN* expression in DiD-haMSCs was still significantly high upon adipogenesis induction. When labeled and unlabeled haMSCs were differentiated under chondrogenic conditions, regional condensations were formed and glycosaminoglycan (GAG) production stained positive for Alcian blue while only faint background Alcian blue staining was observed under control conditions (Fig. 2a), and the cartilage specific *COL2α1* was extensively upregulated during chondrogenesis (Fig. 2b). Taken together, all these in vitro differentiation studies provided evidence that DiD labeling did not alter the stemness or differentiation property of haMSCs.

### Tracking sensitivity of the biofluorescent imaging system was determined in vitro and in vivo

**Fig. 3:**
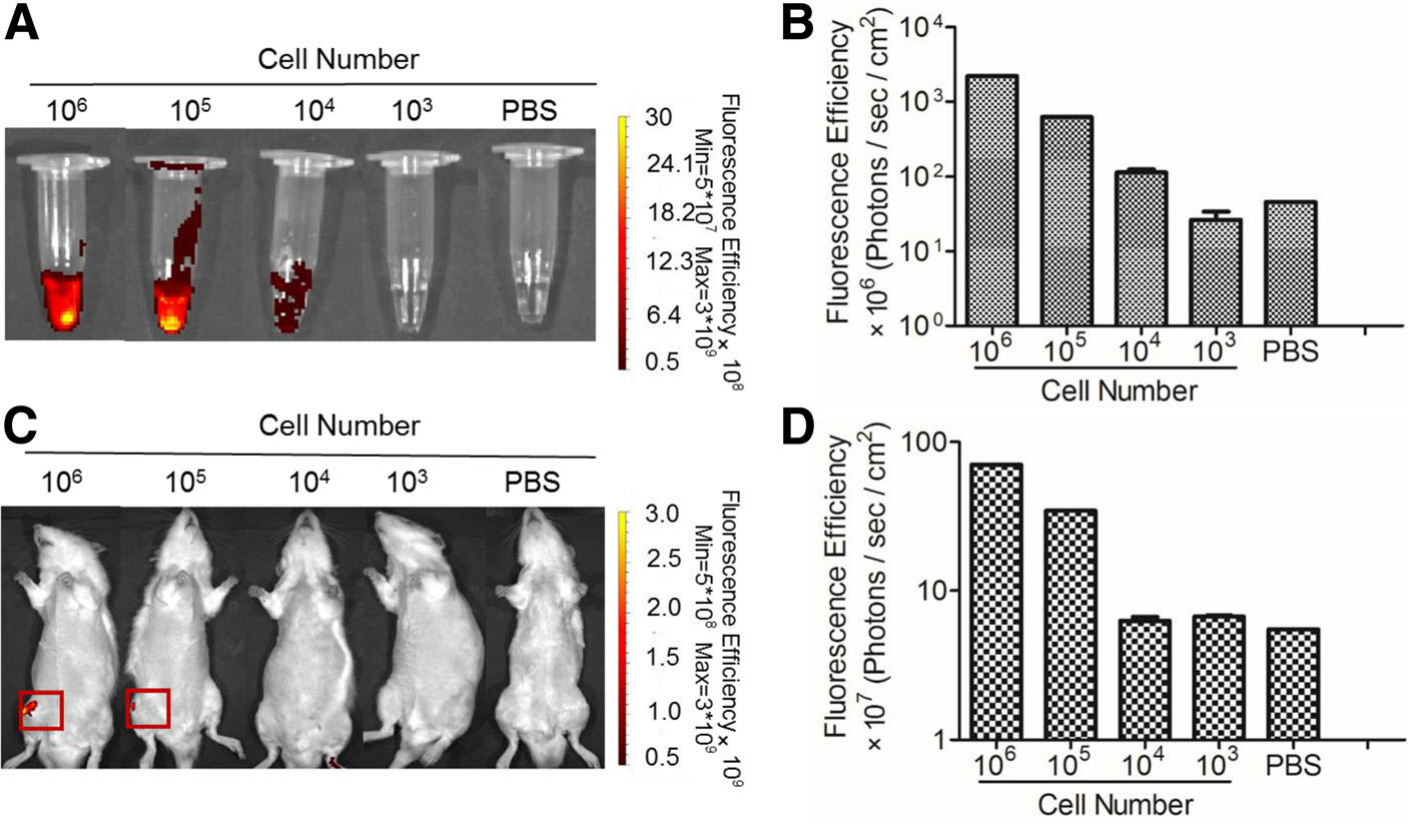
Determination of tracking sensitivity of biofluorescent imaging in vitro and in vivo. **a** Among the serial dilutions, 10^4^ labeled haMSCs displayed a marginal fluorescence signal that could be detected above the phosphate-buffered saline (*PBS*) background. **b** Quantification of the fluorescence signal in **a** showing that the detection threshold in vitro is between 10^3^ and 10^4^ cells (10^7^ and 10^8^ photons/s/cm^2^). **c** Remarkable fluorescent signals were displayed in rats injected with 10^6^ and 10^5^ cells, while signals of 10^4^ cells were undetectable, suggesting that the in vivo detection threshold of DiD-haMSCs was between 10^5^ and 10^4^ cells. **d** Quantification of the fluorescence signal in **c** showing that the detection threshold in vivo is between 10^4^ and 10^5^ cells (8 × 10^7^ and 5 × 10^8^ photons/s/cm^2^). Data were representative of two repetitions (*n* = 2)

To evaluate the detection limit of optical imaging in vitro, serial dilutions of DiD-labeled haMSCs (10^6^, 10^5^, 10^4^, 10^3^, and 0 cells) in 100 μl PBS were placed in Eppendorf tubes and scanned by the IVIS Spectrum system. Positive correlation between fluorescent signals and labeled haMSC numbers were then estimated. The tubes containing 10^6^ and 10^5^ labeled haMSCs demonstrated markedly strong fluorescence while 10^4^ labeled haMSCs displayed marginal fluorescence that could be detected (Fig. 3a). The fluorescence signal reached an undetectable level in the 10^3^ labeled haMSC tube, indicating that the in vitro detection threshold of DiD-haMSCs by the IVIS Spectrum system was between 10^4^ and 10^3^ cells (Fig. 3b). After in vitro detection sensitivity was determined, the in vivo noninvasive detection threshold was also explored by IA injection of DiD-haMSCs (10^6^, 10^5^, 10^4^, 10^3^, and 0 cells) into the knee joint of rats. Results revealed that remarkable fluorescent signals were displayed in rats injected with 10^6^ and 10^5^ cells, while signals in rats injected with 10^4^ cells were undetectable, suggesting that the in vivo detection threshold of DiD-haMSCs by the IVIS Spectrum system was between 10^5^ and 10^4^ cells (Fig. 3c and d).

### Signal of DiD-haMSCs resided in the knee joint

**Fig. 4:**
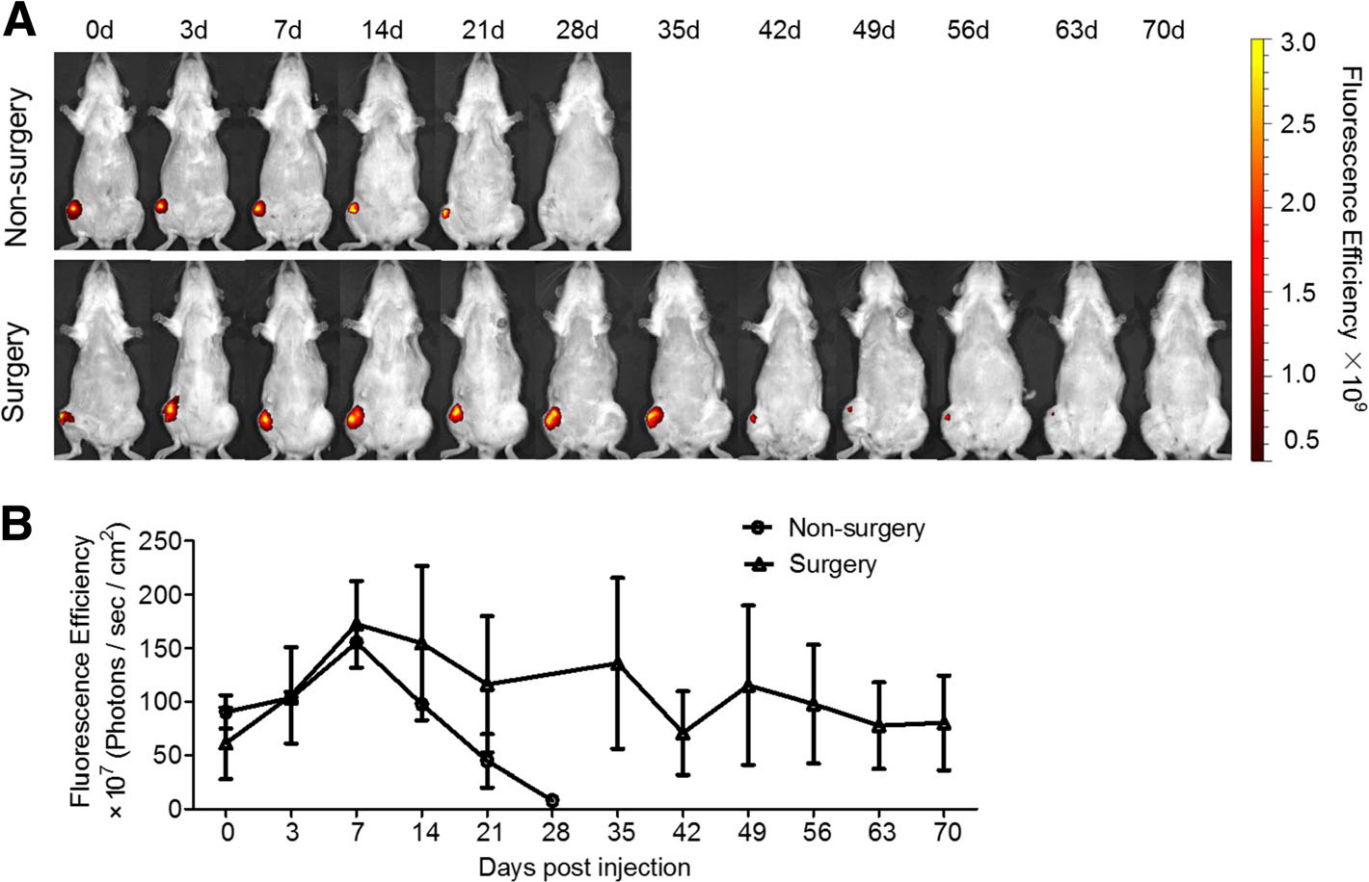
Signal of DiD-haMSCs residing in the knee joint. **a** Detection of florescent imaging of representative rats in both nonsurgery and surgery groups over time. When DiD-haMSCs (2.5 × 10^6^ cells) were injected into the right knee joint of the nonsurgery rats (*top panel*) and surgery rats (*bottom panel*), signals remained relatively stable in surgery joints and became undetectable after 70 days of injection while in the nonsurgery joints the signal diminished rather quickly at day 28. **b** Quantitative fluorescent intensity of the nonsurgery and surgery groups. Data show that the average (*n* = 2) fluorescent intensity appeared to fluctuate as time went on. Signals in the surgery group lasted much longer than in nonsurgery group

After the detection threshold has been determined, we explored the persistence of a therapeutic dose of DiD-haMSCs in the knee joint. When DiD-haMSCs (2.5 × 10^6^ cells) were injected into the right knee joint of normal rats and OA rats, fluorescent signals remained relatively stable in the surgery joints and became undetectable after 70 days following injection, while in the nonsurgery joints the signal diminished rather quickly at day 28 (Fig. 4a). Quantitative fluorescent intensity (*n* = 2) appeared to peak at around day 7 in both the nonsurgery group and surgery group, and then decreased as time went on. However, we suppose that the fluctuation of fluorescence intensity was caused by individual differences and edema created by the invasive procedure (operation and injection) since the cells may proliferate but the fluorescent dye cannot (Fig. 4b).

### Proliferative haMSCs engrafted into rat cartilage

**Fig. 5:**
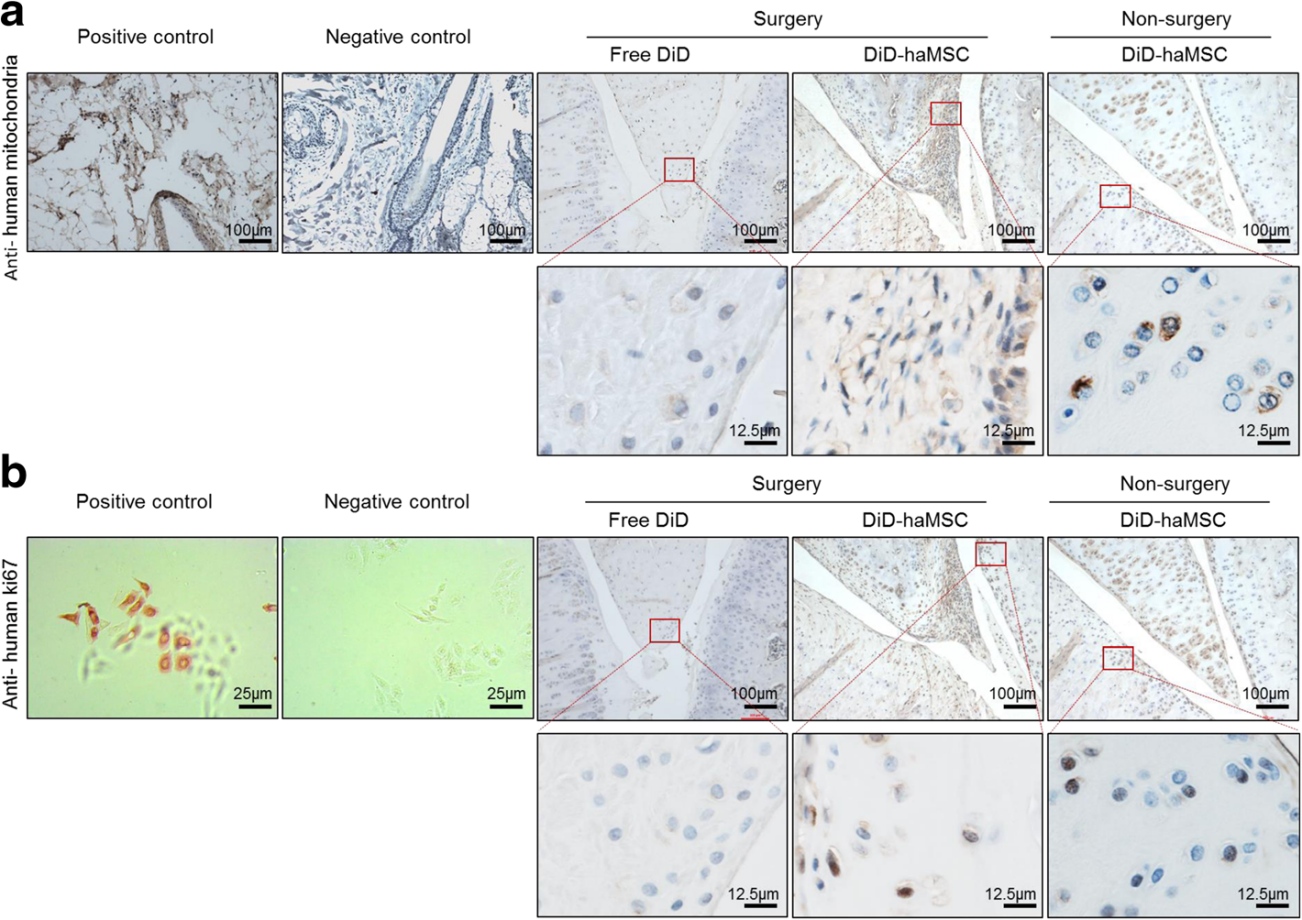
Proliferative human cells were detected in the rat knee joint tissues. **a** Human skin tissues were used for positive (with monoclonal antihuman mitochondria antibody) and negative (IgG isotype) IHC staining controls. Positive signals were detected in both nonsurgery and surgery groups with human adipose-derived mesenchymal stem cell (*haMSC*) treatment, but not in the free DiD group (no haMSC treatment). The human cells were mainly detected in the rat meniscus and cartilage. **b** Human hepatocellular carcinoma cell line HepG2 were used for positive and negative controls with regard to a monoclonal antihuman ki67 antibody IHC staining. Human cells undergoing proliferation were detected in the positive control as well as in the joint tissues of the DiD-labeled haMSC treatment groups, but not in the free DiD group. Samples of the surgery group (*n* = 2) and the nonsurgery group (*n* = 2) were both collected at the end of the experiments (day 70 and day 28), respectively

To understand the fate of the DiD-labeled haMSCs in vivo, we performed immunohistochemical (IHC) staining to visualize human cells in the rat joints. Using monoclonal antihuman mitochondrial antibody, IHC staining demonstrated that human mitochondrial signals were positive in the meniscus and cartilage in the haMSC-treated group and negative in the free DiD group (Fig. 5). In addition, using monoclonal antihuman Ki67 antibody, we visualized human cells undergoing proliferation in the same vicinity to the region where cells also display human mitochondrial identity (Fig. 5). The positive signal for human mitochondria and Ki67 lasted 10 weeks. These data suggested that human MSCs were able to engraft to the meniscus and cartilage in the rat joint, and proliferated in situ.

### No broad organ biodistribution of haMSC by quantitative real-time PCR

**Fig. 6:**
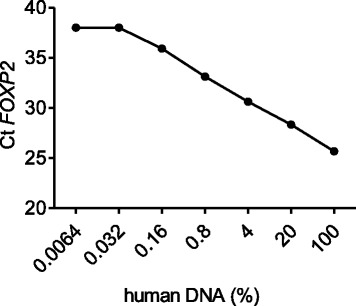
Threshold of human DNA in rat tissue was determined by quantitative real-time PCR using the human-specific *FOXP2* gene probe. DNA from haMSCs were mixed with rat genomic DNA in different concentrations for quantitative PCR showing that 0.032 % human DNA (0.01056 ng human DNA in a total of 33 ng DNA) is the lowest amount detected before reaching the plateau

To further evaluate whether IA injected haMSCs could migrate to distant organs, quantitative real-time PCR detecting human *FOXP2* gene was performed on days 14 and 70. In order to draw a standard curve, DNA from haMSCs was mixed with rat genomic DNA in different concentrations to mimic the real situation (Table 1). We found that the detection threshold was 0.01056 ng human DNA mixed with 32.98944 ng rat DNA, totaling 33 ng DNA in the reaction system (Ct = 38 and reached plateau), indicating 0.032 % human DNA as the lowest amount that could be detected (Fig. 6). After knowing the detection threshold and standard curve, DNA isolated from the heart, liver, spleen, lung, kidney, brain, and knee joint tissues of the haMSC-injected rats at day 14 and day 70 were subjected to quantitative PCR for the human-specific *FOXP2* gene. At each time point, the gene could not be detected in any tissues except for joint muscles, joint ligament, and joint meniscus. Among joint muscles and ligament, human DNA accounted for 0.235 % and 0.035 % of the total DNA content, respectively, in one of three rats sacrificed at 14 days postinjection; at 70 days post-transplantation, human DNA accounted for 0.094 % of the total DNA content of the meniscus in another rat; at both time points, human DNA was not detected in any other organs tested in any animals (Table 2). These results confirmed the in vivo imaging data, indicating that haMSCs remained localized after IA delivery in immunocompetent rats.

**Table 2:** Percentages of human DNA were detected in the indicated organs at the time point indicated

	Heart	Liver	Spleen	Lung	Kidney	Brain	Knee joint				
							Muscle	Meniscus	Synovium	Ligament	Cartilage
14 days postinjection											
Rat 1	–	–	–	–	–	–	0.235 %	–	–	–	–
Rat 2	–	–	–	–	–	–	–	–	–	0.035 %	–
Rat 3	–	–	–	–	–	–	–	–	–	–	–
70 days postinjection											
Rat 4	–	–	–	–	–	–	–	0.094 %	–	–	–
Rat 5	–	–	–	–	–	–	–	–	–	–	–
Rat 6	–	–	–	–	–	–	–	–	–	–	–

All samples were from osteoarthritis rats treated with human adipose-derived mesenchymal stem cells

– indicates under the detection limit

### Therapeutic efficacy of IA injection of haMSCs

**Fig. 7:**
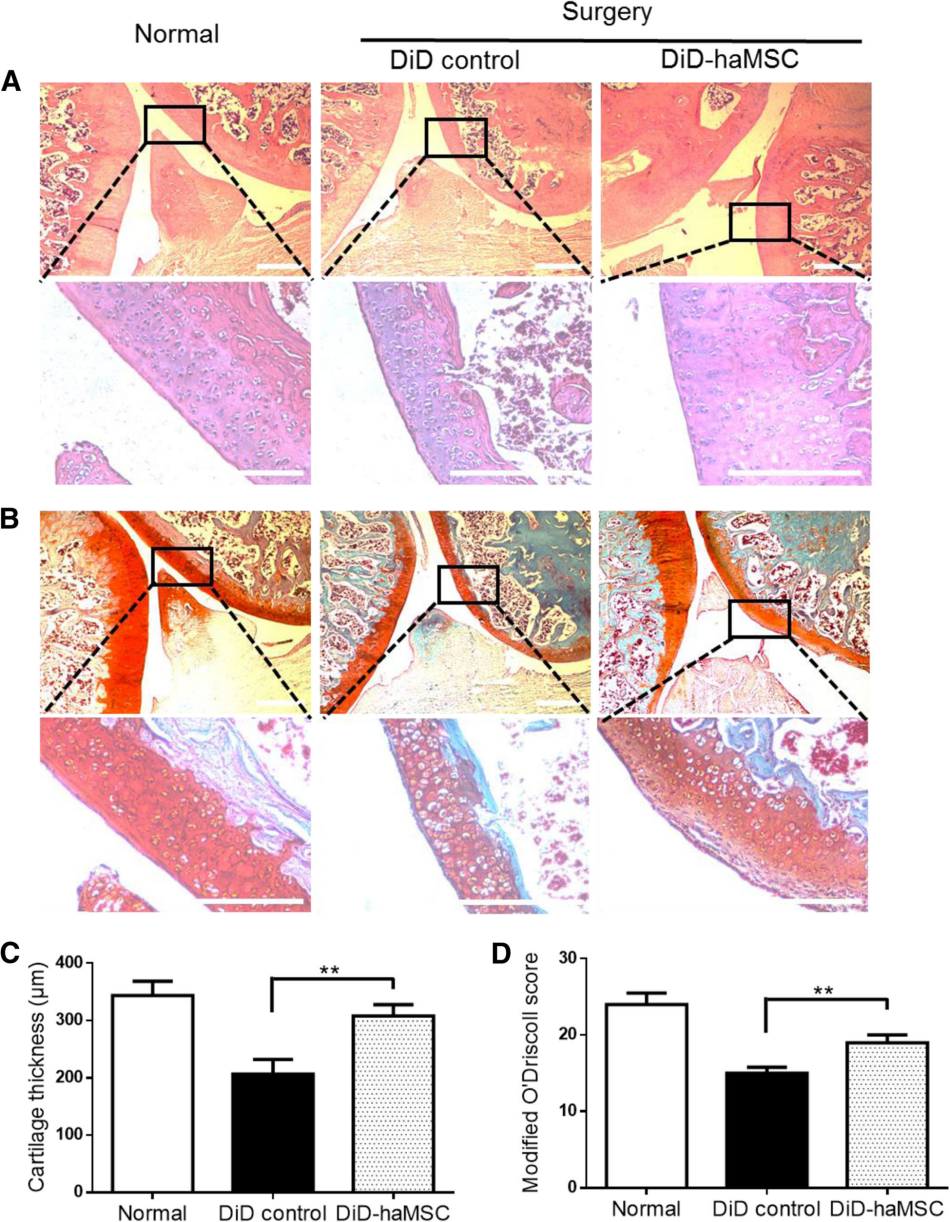
Therapeutic efficacy of IA injection of human adipose-derived mesenchymal; stem cells (*haMSCs*) in the OA model. **a** In the normal group, HE staining showed natural histology of joints with a thick layer of cartilage and subchondral bone, and chondrocytes in organized lacuna (*top panel*, 4×, and *lower panel*, 20×). In contrast, the thickness of the cartilage and especially the subchondral bone in the surgery-induced groups were significantly reduced, and chondrocytes appeared to be more disorganized. haMSC treatment restored the thickness of the cartilage and subchondral bone (*scale bar* = 500 μm). Note that the meniscus in the surgery groups all displayed damage, confirming the successful surgery of meniscectomy for the model creation. **b** Safranin-O/Fast green staining for proteoglycan (PG)/collagen content revealed the significant loss of PG (*red* staining) and increase of fibrillated collagen (*green* staining), a characteristic of degenerative OA phenotypes in the DiD control group compared with the normal group. Treatment with the DiD-haMSCs exhibited increased proteoglycan expression and decreased type I collagen expression (*scale bar* = 500 μm), thus restoring the overall cartilage thickness. **c** Cartilage thickness measured by ImageJ software increased significantly in the haMSC-treatment group compared with the DiD group. **d** The modified O’Driscoll histological score for morphology and structure characteristics quantified the changes, and supported that haMSC treatment showed significant morphological and structural improvement of cartilage. The *P* values were obtained using one-way ANOVA analysis of variance (**P < 0.01). All the experiments were repeated at least two times (*n* = 3)

Since haMSCs after IA injection were mainly concentrated in the joint area, our next step was to try and confirm the therapeutic efficacy at 70 days after the cell therapy to collaborate the biodistribution findings. In the normal group, HE staining showed that the surface of the articular cartilage was smooth and intact while in the DiD control group (surgery-induced OA without cell treatment), articular cartilage exhibited rough borders. Safranin-O/Fast green staining clearly revealed the progression of degenerative OA phenotypes in the free DiD control group compared with the normal group by increased type I collagen expression. In the group with the DiD-haMSC treatment, the joints of affected animals displayed a continuous cartilage surface with few fissures (Fig. 7a), exhibiting decreased type I collagen expression and increased proteoglycan expression (Fig. 7b). The modified O’Driscoll histological score revealed that the DiD-haMSC treatment showed significantly higher scores or better tissue preservation compared to the DiD control group (Fig. 7c). Cartilage thickness also increased significantly in the DiD-haMSC treatment group compared with the free DiD group (Fig. 7d). Taking all data together, it was clear that DiD-labeled haMSCs were able to prevent the cartilage degeneration in surgery-induced OA animal models as seen in similar haMSC efficacy studies without DiD labeling; therefore, the results of MSC persistence and biodistribution from this study becomes very much relevant to the mode of action and therapeutic effect of haMSCs for OA treatment.

## Discussion

A number of previous investigations have reported the efficacy of IA administration of MSCs for the treatment of OA [1, 15, 16]; however, the fate of injected MSCs remains unknown and we do not know how long MSCs have to be in the joint in order to see the efficacy. In our study, we tried to understand the fate and biodistribution of the haMSCs in the context of efficacy. Our data show that haMSCs can be found in the rat joints via intra-articular injection for the duration of the efficacy period, as long as 70 days. We have also found that the MSCs have a higher homing tendency to the injured joint than the normal joint (persisted longer). Our data are consistent with the study in nonOA SCID mice [17]. However, SCID mice were immune-deficient lacking both B and T lymphocytes [18] while most of the OA patients in the clinic would have normal immune function. Thus, MSC biodistribution in immunocompetent OA animal models is more relevant to the clinic. Moreover, the life span and migration rate of MSCs in OA joints and normal joints may be different due to the OA inflammatory microenvironment, and secretion of inflammatory factors may attract MSCs homing to and staying in the injury area [19], which is supported by our findings.

The 10-week persistence of the MSCs in the OA joint coincides with the time for which we observed efficacy, strongly suggesting that OA efficacy by MSCs requires a persistent cell activity rather than transient activity. The finding that xenogeneic haMSCs engrafted into rat meniscus and cartilage but not into other distant organs is very intriguing and provides direct evidence that MSCs are most likely conducting or initiating homeostasis and tissue regeneration locally rather than systemically. Another important finding, which to our knowledge is perhaps for the first time, is that antihuman ki67 signals have been found in rat meniscus and cartilage confirming in vivo proliferation of those injected haMSCs (Fig. 5). This supports the concept of cell therapy as a living drug. Formerly, CFDA-SE-labeled 7.0 × 10^6^ human MSCs were injected into the OA joint of immunocompetent Hartley strain guinea pigs, and labeled cells could be detected by histology at least 5 weeks afterwards in the OA cartilage [7]. Another investigation also found that after injection of 1.0 × 10^6^ GFP-transduced MSCs into caprine OA joints, GFP-positive cells were identified primarily at the surface of neomeniscal tissue 6 weeks after injection [1]. Nonetheless, our data not only provide data for the lifespan of injected MSCs but also in vivo biodistribution, with the conclusion that MSCs injected via IA remained localized and did not migrate to other distant organs and sites at the detection level. Another point provided by our study is that most of the previous in vivo tracking studies only focused on the use of tracking methodology [6, 11, 17, 20]; however, investigation of the tracking technology itself, especially the sensitivity or limitation of each methodology, has not been fully explored. The present study focused not only on the validity of biofluorescent imaging technology but also on the sensitivity of the technology to understand the underlying biology. We found that by using the IVIS Spectrum system the detection threshold of DiD labeling was 10^4^ cells and 10^5^ cells in vitro and in vivo, respectively. Although the sensitivity could be further improved, it is the first time that the number of MSCs detected in the joints of OA animal models over time has been revealed.

In the present study, we have also obtained biodistribution data. The accurate and sensitive human *FOXP2* sequence was used to quantify human DNA in the xeno background and to avoid possible artifacts due to potential rat genome interference. *FOXP2* was the first gene that was determined to be related to development of human speech and language [21]. Due to its high conservation, the *FOXP2* gene was identified as a human-specific reporter gene in human genomic DNA [22–24]. We found that after 14 days of administration, the human *FOXP2* sequence could be detected in the joint ligament and muscles, whereas after 70 days of injection the human-specific *FOXP2* sequence could be detected in the meniscus of the joint in some of the animals. We did not get these detection results consistently across all three animals tested, partly because of the low amount of human DNA present when considering the amount of disparity in human cells in the animal organ tissues. Human DNA had not been detected in the cartilage, but IHC staining identified human signals in the cartilage (Fig. 5). It is possible that human DNA was missed when partial organ/tissue were grinded for DNA isolation. The detection of human cells in only very limited and joint-related organs and tissues actually suggests the validity of the qualitative method.

There are still some limitations in this study. For example, although rats were chosen for this study and other OA preclinical experiments [6, 25], rats are not upright walking animals and the anatomy of rat knee joints is not close to those of humans. Furthermore, in vivo kinetics of the IA injection of MSCs has been demonstrated, but the sensitivity of biofluorescent imaging needs further improvement if fewer than 10^4^ cells need to be detected. More sensitive technologies, such as magnetic resonance imaging or radiolabeled MSCs, should be investigated. Building on this study, it may be possible to closely establish the PK/PD relationship with more precise technologies and time points to elucidate the mechanism and cell activity required for efficacy in treating OA patients.

## Conclusion

The current study demonstrated PK/PD of labeled haMSCs after IA injection in an immunocompetent surgery-induced OA rat model. We found that proliferation and differentiation of haMSCs were not affected by the labeling with DiD dye, and the detection thresholds of the labeled cells in vitro and in vivo were determined to be 10^4^ and 10^5^ cells, respectively. Moreover, fluorescent signals in OA rats lasted for about 10 weeks in the knee joint at the same time as efficacy was observed. Furthermore, human-specific markers could only be detected in the joint ligament, meniscus, and muscles adjacent to the joint, not in other distant organs. Although more sensitive methods may need to be investigated, the current results suggest that local persistence of adipose-derived human mesenchymal stem cells may be important for their functions on promoting joint regeneration to be exerted, further supporting the safety and feasibility of IA injection of MSCs in the treatment of OA.

## Abbreviations

CFDA-SE: Carboxyfluorescein diacetate succinimidyl ester
Col2α1: Collagen type 2 alpha 1
CT: Cartilage thickness
DiI: Dialkylcarbocyanines
DNA: Deoxyribonucleic acid
EDTA: Ethylenediaminetetraacetic acid
FOXP2: Forkhead box P2
GAG: Glycosaminoglycan
GFP: Green fluorescent protein
HA: Hyaluronic acid
haMSC: Human adipose-derived mesenchymal stem cell
HE: Hematoxylin and eosin
IA: Intra-articular
IHC: Immunohistochemical
MOA: Mode of action
MRI: Magnetic resonance imaging
MSC: Mesenchymal stem cell
OA: Osteoarthritis
OD: Optical density
PBS: Phosphate-buffered saline
PCR: Polymerase chain reaction
PD: Pharmacodynamics
PK: Pharmacokinetics
ROI: Region of interest
SCID: Severe combined immune deficiency
VAS: Visual analogue scale
WOMAC: Western Ontario and McMaster Universities Osteoarthritis Index
